# Genomic Characteristics and Comparative Genomics Analysis of the Endophytic Fungus *Paraphoma chrysanthemicola* DS-84 Isolated from *Codonopsis pilosula* Root

**DOI:** 10.3390/jof9101022

**Published:** 2023-10-16

**Authors:** Wenbin Sun, Min Feng, Ning Zhu, Feifan Leng, Mingjun Yang, Yonggang Wang

**Affiliations:** School of Life Science and Engineering, Lanzhou University of Technology, Lanzhou 730050, China; sunwb@lut.edu.cn (W.S.); 212105500037@lut.edu.cn (M.F.); 221081700010@lut.edu.cn (N.Z.); lengffphils@lut.edu.cn (F.L.); yangmj@lut.edu.cn (M.Y.)

**Keywords:** *Paraphoma chrysanthemicola*, genome and comparative genomics analysis, endophytic fungus, *Codonopsis pilosula* root

## Abstract

*Paraphoma chrysanthemicola* is a newly identified endophytic fungus. The focus of most studies on *P. chrysanthemicola* has been on its isolation, identification and effects on plants. However, the limited genomic information is a barrier to further research. Therefore, in addition to studying the morphological and physiological characteristics of *P. chrysanthemicola*, we sequenced its genome and compared it with that of *Paraphoma* sp. The results showed that sucrose, peptone and calcium phosphate were suitable sources of carbon, nitrogen and phosphorus for this strain. The activities of amylase, cellulase, chitosanase, lipase and alkaline protease were also detected. Sequencing analysis revealed that the genome of *P. chrysanthemicola* was 44.1 Mb, with a scaffold N50 of 36.1 Mb and 37,077 protein-coding genes. Gene Ontology (GO) annotation showed that mannose-modified glycosylation was predominant in monosaccharide utilisation. The percentage of glycoside hydrolase (GH) modules was the highest in the carbohydrate-active enzymes database (CAZy) analysis. Secondary metabolite-associated gene cluster analysis identified melanin, dimethylcoprogen and phyllostictine A biosynthetic gene clusters (>60% similarity). The results indicated that *P. chrysanthemicola* had a mannose preference in monosaccharide utilisation and that melanin, dimethylcoprogen and phyllostictine A were important secondary metabolites for *P. chrysanthemicola* as an endophytic fungus.

## 1. Introduction

Endophytic fungi live within about 300,000 plant species on Earth without causing any apparent symptoms of disease [1]. Endophytic fungi are mainly members of the Ascomycota and some taxa of the Basidiomycota, Zygomycota and Chytridiomycota [2,3]. Endophytic fungi have a symbiotic relationship with hosts to obtain nutrients for survival [4]. This symbiosis allows endophytic fungi to enhance the biomass of the host, decrease water consumption and increase tolerance to abiotic and biotic stresses [5]. These phenomena are supported by the fact that endophytic fungi produce secondary metabolites (auxin, gibberellins, cytokinins, indole-3-acetic acid, polyketides and non-ribosomal peptides) [6]. The synthesis of secondary metabolites is influenced by the genotype of the endophytic fungi [7,8]. The analysis of biosynthetic gene clusters using whole-genome information is an important research tool for the characterisation of fungal genotypes.

In the genomic era, the genotypes of endophytic fungi can be well understood by conducting genome-wide studies. The ability of endophytic fungi to interact with plants has been linked to the expression of specific genes or clusters of genes [9]. The analysis of endophytic fungal genomic information uses genetic information technologies, including gene prediction, non-coding RNA prediction, gene annotation and advanced gene annotation. Tools include Genemark-ES, RNAmmer, BLAST and antiSMAH, as well as the non-redundant protein sequence database (NR), uniprot, GO, Kyoto Encyclopedia of Genes and Genomes (KEGG) and CAZy databases [10]. Genome mining can be used for a variety of purposes, including pathway prediction for specific or novel secondary metabolites and homolog searches for pathway engineering [11]. The biological functions of secondary metabolites identified from plant endophyte genome mining include anti-cancer (tumour), anti-Alzheimer’s disease, anti-viral, anti-bacterial, anti-fungal and anti-insect. The identification of genes for the biosynthesis of a large number of secondary metabolites isolated from endophytic fungi provides an opportunity to explore the genetic potential of production strains to discover new secondary metabolites and to enhance the production of secondary metabolites through metabolic engineering, leading to new and more affordable pharmaceutical and food additives [12].

*Paraphoma chrysanthemicola* (*P. chrysanthemicola*) is a common soil-borne fungus [13]. *P. chrysanthemicola* was originally isolated from diseased plant root tissue and was considered a pathogen [14,15]. However, *P. chrysanthemicola* has also been shown to increase plant biomass and improve tolerance to nematodes, salt stress, drought stress and heavy metals [16,17,18,19,20,21]. Morphological and molecular markers are mainly used to identify *P. chrysanthemicola* [15]. Morphologically, *P. chrysanthemicola* is clearly characterised by radial growth, pigment secretion, mycelial diaphragm and chlamydospore production [22]. The original classification of *P. chrysanthemicola* in taxonomy placed it in the *Paraphoma* section of the genus *Phoma* and, subsequently, *Paraphoma* was introduced as a new genus using phylogenetic analysis [23]. There are currently three genomes of the genus *Paraphoma* in the NCBI database, *Paraphoma* sp. B47-9 (GCA_001748405.1) and *P. chrysanthemicola* (GCA_020744225.1 and GCA_020744215.1) (https://www.ncbi.nlm.nih.gov/; accessed on 8 October 2023). Most studies on *P. chrysanthemicola* have focused on its isolation, identification and effects on plants. Its genome has not been analysed, even if it has been sequenced. In this study, we sequenced the *P. chrysanthemicola* strain isolated and identified from *Codonopsis pilosula*, the sequence of which has been uploaded to GenBank, and analysed its genomic characteristics by integrating the available genomic data of this strain. This provides a theoretical basis for future deep mining and exploitation of the fungal genes of this species as an endophyte.

## 2. Materials and Methods

### 2.1. Plant Source, Strain Culture and Maintain

The *C. pilosula* seeds used in this research were purchased from Angelica Research Institute in Minxian County, Gansu Province, China, and planted in the experimental base in accordance with the national guidelines.

The test strain, *P. chrysanthemicola* (DS-84), was isolated from the roots of *Codonopsis pilosula* and maintained in our laboratory. A 7 mm diametrical portion of a fungal colony was cut with a cork borer and inoculated into the centre of a sterilised potato dextrose agar (PDA) plate in a 9 cm diameter Petri dish at 25 °C. 

### 2.2. Observation of Strain Morphology by Scanning Electron Microscopy (SEM)

The observation of morphological and cultural characteristics was in accordance with previous studies [15,24]. Fresh fungal blocks were washed with phosphate-buffered saline and treated with 2.5% glutaral solution at 4 °C. After rinsing with phosphate buffer (0.1 M; pH 7.4) for 2 h, the samples were dehydrated using a series of increasing concentrations (30%, 50%, 70%, 80%, 90%, 95%, 100% and 100%) of ethanol solution. The process of critical point drying, mounting and gold spraying was completed last. The samples were then observed and photographed using a SEM (SU8100, HITACHI, Tokyo, Japan).

### 2.3. Fourier Transform Infrared (FTIR)

The mycelium was dried in a vacuum freeze-dryer, powdered in liquid nitrogen and stored at −20 °C. Frozen samples were thawed at room temperature prior to each FTIR measurement. The infrared spectrum of the samples was collected using the attenuated total reflection (ATR) mode on a Perkin Elmer Spectrum 100 FTIR spectrometer (Perkin Elmer, Norwalk, CT, USA) equipped with a universal ATR accessory. ATR data were obtained from the diamond/ZnSe crystal of the ATR unit equipped with a 45-degree angle of incidence. A finely ground sample powder was placed flatwise in a groove in the diamond/ZnSe crystal and pressed firmly to measure the infrared spectrum. Test conditions: spectral resolution 4 cm^−1^ and measuring range 800–4000 cm^−1^. Twenty-three scans were accumulated and automatically smoothed (Perkin-Elmer, Waltham, MA, USA). Interference from H_2_O and CO_2_ was subtracted in real-time during the scan. An empty scan without any sample was set as background and a background scan was performed for every 5 samples measured. Spectral data were recorded and analysed using Perkin Elmer’s Spectrum One software (version 6) and plotted using R’s ggplot2 package (version 3.4.3).

### 2.4. Mycelial Fatty Acid Fraction

After grinding, the mycelium was mixed with CHCl_3_ and 1 mol/L NaOH/CH_3_OH solution, then 2 mol/L NaOH/CH_3_OH solution was added and mixed for 10 min at room temperature; finally, double-distilled water was added and shaken for 1 min and then allowed to stratify. The bottom layer was removed and dehydrated with anhydrous Na_2_SO_4_. An amount of 0.5 μL of sample was aspirated into a gas chromatograph (8860 GC system, Agilent, Santa Clara, CA, USA) equipped with a flame ionisation detector (FID) and a capillary column (Agilent DB FFAP, 0.25 mm × 0.25 um × 30 m, Santa Clara, CA, USA). Helium was used as the carrier gas. The column temperature was increased from 70 to 2000 °C at a rate of 10 min. Fatty acid characterisation: the retention time of the control standard was used for characterisation (standard purchased from Sigma, St. Louis, MI, USA). Quantification of fatty acids: the percentage content was determined by area normalisation and the concentration was determined from the standard curve.

### 2.5. Molecular Identification of Fungi

DNA was extracted using a fungal genomic DNA extraction kit (Beijing Solabao Technology Co., Ltd., Beijing, China). Three pairs of different primers (Table S1) (ITS1 and ITS4, NS1 and NS4, LROR and LR5) were used to amplify the internal transcribed spacer (ITS) of ribosomal DNA, including the 5.8S rRNA gene, nuclear ribosomal small subunit (SSU) and large subunit (LSU) by PCR [25,26]. The amplification products of the PCR were observed on a 1% agarose gel. PCR products were sequenced by Sangon Biotech (Shanghai, China). The nucleotide sequence similarities were obtained by BLAST (http://blast.ncbi.nlm.nih.gov/blast/Blast.cgi; accessed on 11 October 2023). Multiple sequence alignments were performed using ClustalW (version 2.1). Phylogenetic trees were constructed using the neighbour-joining method in MEGA6 software (http://www.megasoftware.net; accessed on 11 October 2023).

### 2.6. Effects of Carbon and Nitrogen Sources on Fungi Growth

Preserved strains were inoculated onto PDA and incubated for 10 d at 25 °C in the dark, then the edge of the colony was punctured with a cork borer (7 mm diameter) for inoculation into Czapek–Dox medium. Different carbon sources were obtained by replacing glucose (control) with equal amounts of mannitol, sucrose, fructose, lactose, maltose and soluble starch as the basal medium. Different nitrogen source media were obtained by replacing sodium nitrate (control) with equal amounts of inorganic nitrogen (potassium nitrate, calcium nitrate and ammonium sulphate) and organic nitrogen (peptone and yeast powder). Inorganic phosphorus (calcium phosphate) and organic phosphorus (lecithin) were used in equal amounts instead of dipotassium hydrogen phosphate (control) to obtain different phosphorus source media. Colony diameters were measured at 36 h intervals and the average mycelial growth rate was calculated. The strains were fermented in shake flasks for 10 d. The mycelium was collected and dried at 80 °C to obtain the dry weight. Three replicates were performed for each treatment.

### 2.7. Characterisation of the Enzyme Produced by the Fungi

The enzymatic properties of the strains were assessed by the substrate used or dissolved by the fungi in the agar medium [27]. One-week-old 7 mm diameter portions of a fungal colony obtained from the PDA were inoculated individually into the appropriate media, with three replicates for each colony, and after 3–7 d of incubation at 25 °C, the presence of hyaline or discoloured areas around the fungal colonies were used as a qualitative assay for enzyme production; fungus-free agar plates with the substrate were used as a negative control. Enzyme activity was expressed as the difference between the diameter of the transparent circle and the diameter of the colony. Amylase activity was measured in glucose–yeast extract–peptone agar (GYP) medium containing 0.2% starch, cellulase activity was measured in GYP medium containing 0.5% Na-carboxymethylcellulose and lipase activity was measured in peptone agar medium containing 1% Tween 20. The alkaline protease, fibrinolytic enzyme, catalase and chitosanase activities were determined in alkaline protease-identification medium (1 L of medium contains 10.0 g peptone, 1.0 g glucose, 0.1 g CaCl_2_, 0.1 g tyrosine, 5.0 g casein and 22.0 g agar, with pH 8.5–9.0), fibrinolytic enzyme screening medium (1 L of medium contains 15.0 g maltose, 10.0 g casein, 15.0 g NaCl and 20.0 g agar, with pH 7.2–7.4), catalase-screening medium (1 L of medium contains 200.0 g potatoes, 20.0 g glucose, 3.0 g MgSO_4_·7H_2_O, 5.0 g KH_2_PO_4_, 0.01 g vitamin B1, 20.0 g agar and 5.0 mmol hydrogen peroxide, with neutral pH, sterilised and cooled to 50 °C) and chitosanase screening medium (1 L of medium contains 10.0 g water-soluble chitosan, 5.0 g (NH_4_)_2_SO_4_, 1.0 g enzyme extract, 20.0 g K_2_HPO_4_·3H_2_O, 1.0 MgSO_4_·7H_2_O, 5.0 g NaCl and 20.0 g agar, with pH 6.5.).

### 2.8. Fungal Genomics Analysis

DNA was extracted using a fungal genomic DNA extraction kit (Beijing Solabao Technology Co., Ltd., Beijing, China). Qubit (Thermo Fisher Scientific, Waltham, MA, USA) and Nanodrop (Thermo Fisher Scientific, Waltham, MA, USA) were used to measure DNA quality. Genomic DNA was fragmented and end-repaired using G-tubes (Covaris, Woburn, MA, USA). Repaired DNA was selected using a Blue Pippin system to construct SMRTbell DNA libraries with fragment sizes of >10 kb according to the manufacturer’s specifications (PacBio, Menlo Park, CA, USA). Library quality was determined using a Qubit 2.0 Fluorometer (Life Technologies, CA, USA) and the average fragment size was estimated using a Bioanalyzer 2100 (Agilent, Santa Clara, CA, USA). Pacific Biosciences Sequel (PacBio, Menlo Park, CA, USA) was used for SMRT sequencing according to standard protocols. Consecutive long reads were obtained from three SMRT sequencing runs. Reads with mass values greater than 0.75 and greater than 500 bp were combined into one data set. Random errors in long seed reads (seed length threshold of 6 Kb) were then corrected using the hierarchical genome assembly process (HGAP) by aligning shorter reads from the same library to them [28]. The resulting corrected pre-assembled reads were used for de novo assembly using the Celera Assembler (version 8.3) with an overlap–layout–consensus (OLC) strategy [29,30]. No quality values were used in the assembly process, as SMRT sequencing has minimal quality variation across reads. The Quivier consistency algorithm was used to verify the quality of the assembly and to determine the final genome sequence [28]. The reads were further assembled into scaffolds using Multi-CSAR (https://github.com/ablab-nthu/Multi-CSAR; accessed on 11 October 2023) with reference genomes of *Paraphoma* sp. B47-9 (GCA_001748405.1, GCA405) and *P. chrysanthemicola* (GCA_020744225.1 and GCA225) [31]. The GCA405 genome was also further assembled.

Genes were de novo and homologously predicted using GeneMark-ES (version 4.0) (reference is the GCA225 protein sequences, https://www.ncbi.nlm.nih.gov/data-hub/genome/GCA_020744225.1/; accessed on 11 October 2023) [32,33]. Scattered repeats were identified using RepeatMasker (version 4.1.1) with the database built by RepeatModeler (version 2.0.3), and tandem repeats were predicted using Tandem Repeats Finder (version 4.09) [34,35,36]. Non-coding RNAs were predicted using RNAmmer (version 1.2), tRNAscan-SE (version 2.0.11) and Infernal (version 1.14) [37,38]. Protein sequences were compared with the non-redundant (NR), uniprot and CAZy databases using Diamond (version 2.1.8) for protein annotation (protein annotation results are the intersection of the annotated NR and uniprot databases), GO annotation and carbohydrate-related enzyme annotation [39,40,41,42]. KEGG annotation was performed using eggNOG (version 6.0) [43]. Secondary metabolic gene clusters were predicted using antiSMASH 6.0 (https://fungismash.secondarymetabolites.org/#!/start; accessed on 11 October 2023) with >60% similarity [44]. The analysis of ks/ka values for secondary metabolic gene clusters was performed in the three genomes using WGDI (version 0.6.2) [45]. Mummer (version 3.23) was used to analyse the homology of genomes DS-84, GCA225 and GCA405. The results were plotted using the R language packages ggplot2 (version 3.4.3), circlize (version 0.4.15), simplifyEnrichment (version 1.10) and TBtools (version 2.012) [46,47,48,49]. The results were taken from the intersection of the three genomes DS-84, GCA225 and GCA405 after comparative analysis. The sources of all reagents and chemicals used in this study are listed in Table S2.

## 3. Result

### 3.1. Strain Identification

**Figure 1:**
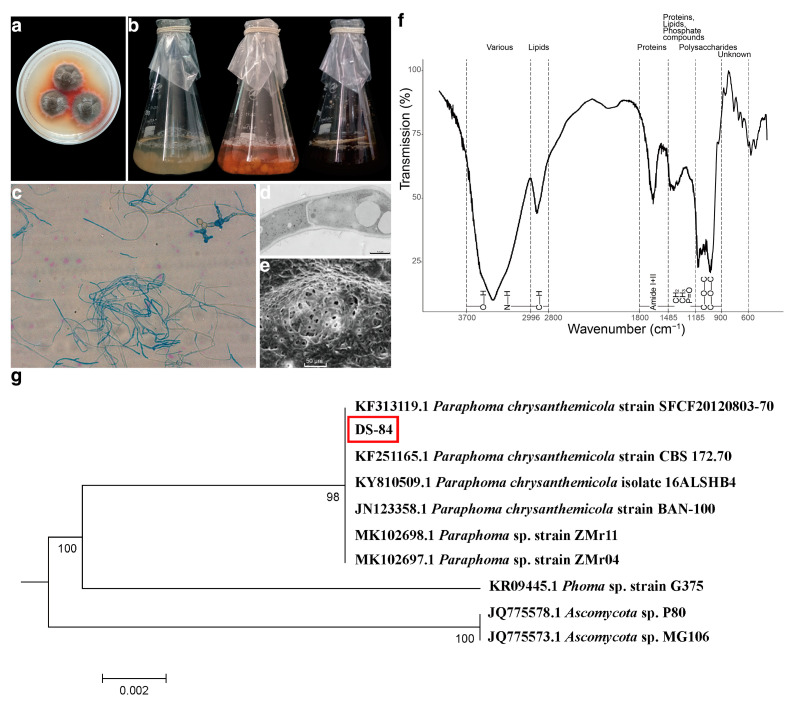
Evidence for the identification of DS-84 strain. Colony morphology of DS-84 shown in solid and liquid PDA medium (**a**,**b**). Optical microscopy of microstructure of mycelium (**c**). Transmission and scanning electron microscopy of sub-microstructure of mycelium (**d**,**e**). Dotted lines mark the wavenumber ranges of dominating chemical compounds (**f**). DS-84, highlighted with red box, phylogenetic tree formed with 1000 bootstrap replicates based on rDNA-ITS sequences (**g**).

The DS-84 strain isolated from the root of *Codonopsis pilosula*. The DS-84 strain formed dense and carpet-like colonies. The surface was rough with a white edge. This strain produced an orange–red pigment (Figure 1a). The increase in pigment secretion was more clearly observed over time through the change in the colour of the liquid medium (Figure 1b). At the same time, the mycelium changed from white to brown. The mycelium was septate and laterally branched (Figure 1c). Chlamydospores and chains of chlamydospores were occasionally observed, which were darkly stained, globose or subglobose, and formed by the modification of the hyphal cell (Figure 1c). SEM revealed a fungus with a rough surface and pores of varying sizes (Figure 1d). Transmission electron microscopy revealed thick cell walls and vesicles (Figure 1e).

The structural features of the compounds of the DS-84 mycelium were detected by FTIR spectroscopy to form a fingerprint profile (Figure 1f). The structural features of the absorbing fraction in the fungal mycelium were labelled according to the steps proposed in the methods section, as well as by comparing the similarity to other mycelium structural features. The results showed that the C-H, CH_2_, CH_3_ and P=O structures all had absorption peaks in the lipid signature region. This was similar to the analysis of mycelial lipid fractions. The variation in fingerprint profiles was also generally consistent with the variation in the FTIR spectra of other fungal hyphae.

The mycelium contains nine fatty acids, in which the relative content of unsaturated fatty acids was 75.19%, about 4.26 times that of saturated fatty acids, which were mainly monounsaturated (Table 1). Among the nine fatty acids, the highest relative content of C18 acid was 63.87%, followed by C16 acid. Proportions of 31.46% and 29.31% of the C18 acids were oleic acid (C18:1n9c) and linoleic acid (C18:2ω6), respectively.

The strains were further subjected to molecular identification by analysing the ribosomal internal transcribed spacer (ITS) region. DNA isolated from the strain was amplified with ITS1 and ITS4 primers and specific amplicons of about 560 bp were generated (Supplementary Figure S1a). The results of pairwise comparison of DS-84 ITS sequences are depicted in Figure 1g. The best match results from *Paraphoma* genus were 99.27% to 100%. DS-84, MK102698.1 (*Paraphoma* sp. strain ZMr11), MK102697.1 (*Paraphoma* sp. strain ZMr04) and KF251165.1 (*Paraphoma chrysanthemicola* strain CBS 172.70) formed a group with strong bootstrap support of 100%. Strains of DS-84 and JN123358.1 (*Paraphoma chrysanthemicola* strain BAN-100) formed a group with a bootstrap support of 99.82%. DS-84 clustered together with KF313119.1 (*Paraphoma chrysanthemicola* strain SFCF20120803-70) with a bootstrap support of 99.27%. The results of the rDNA-SSU and rDNA-LSU sequences were consistent with ITS (Supplementary Figures S1b–d and S2–S4). All these results suggested that the strain DS-84 was *Paraphoma chrysanthemicola*, hence the name *Paraphoma chrysanthemicola* DS-84.

### 3.2. Effect of Different Carbon, Nitrogen and Phosphorus Sources on the Morphology of DS-84

Different sources of carbon, nitrogen and phosphorus gave different colours to the DS-84 colonies (Supplementary Figure S5). The colour of the colonies in the glucose, mannitol, fructose, sucrose, starch, KNO_3_ and Ca(NO_3_)_2_ groups was dark grey. Maltose, lactose, (NH4)_2_SO_4_, peptone, yeast powder, KH_2_PO_4_ and lecithin gave the DS-84 colonies a white colour. (NH_4_)_2_SO_4_ and KH_2_PO_4_ also caused the fungus to produce an orange–red pigment. 

**Figure 2:**
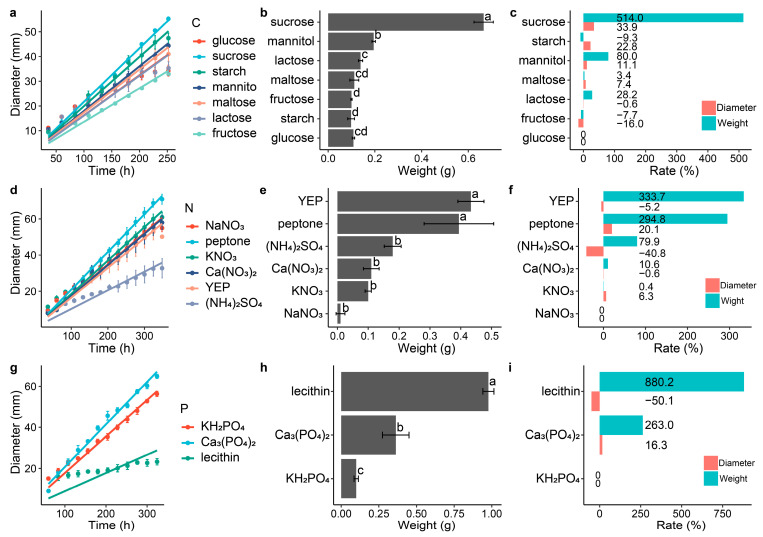
Effect of different carbon, nitrogen and phosphorus sources on the growth of strain DS-84. Strain DS-84 grown on different carbon sources (**a**–**c**). Strain DS-84 grown on different nitrogen sources (**d**–**f**). Strain DS-84 grown on different phosphorus sources (**g**–**i**). Change in colony diameter is depicted with incubation time (**a**,**d**,**g**). Effects of different carbon, nitrogen and phosphorus sources on the mycelial weight of strain DS-84 are compared (**d**–**f**). Letters indicate significant differences (*p* < 0.05). Effect of different carbon, nitrogen and phosphorus sources on colony diameter and mycelial weight of strain DS-84 relative to glucose, NaNO_3_ and KH_2_PO_4_ (**c**,**f**,**i**).

Different carbon, nitrogen and phosphorus sources had different effects on the growth of the DS-84 (Figure 2). Compared with the control group, disaccharides in the carbon source promoted the accumulation of biomass (Figure 2a–c). Among them, sucrose promoted biomass accumulation and colony growth most significantly, increasing the colony growth rate by 33.9% and weight by 5.1 times. Lactose had the least effect on biomass accumulation (28.2%) and also reduced the increase in colony diameter (−0.6%). Among the monosaccharides and derivatives, mannitol significantly promoted colony weight (80%) and diameter (22.8%), while fructose inhibited DS-84 growth (weight −7.7% and diameter −16.0%). The main carbon source of the PDA medium, starch, inhibited the material accumulation of DS-84 (−9.3%), but increased the colony diameter (22.8%). The accumulation of DS-84 material was promoted by different nitrogen sources, with the organic nitrogen sources yeast powder (YEP) and peptone increasing colony weight by 3.3 and 2.9 times, respectively (Figure 2d–f). Meanwhile, YEP decreased the colony diameter by 5.2% and peptone increased the colony diameter by 20.1%. Inorganic nitrogen sources of ammonium sulfate and calcium nitrate reduced the colony diameter by 40.8% and 0.6%. Similar to the nitrogen source, different phosphorus sources promoted the accumulation of DS-84 biomass (Figure 2g–i). Lecithin and calcium phosphate increased the colony weight by 8.8 and 2.6 times, respectively. However, lecithin inhibited the increase in colony diameter (−50.1%), while calcium phosphate promoted the increase in colony diameter (16.3%).

This study also characterised some enzyme-producing properties of strain DS-84 (Table 2). The results showed that DS-84 possessed amylase, cellulase, alkaline protease and chitosanase activities. Lipase, fibrinolytic enzymes and catalase produced a negative result. The media showing positive enzyme activity relative to the control were more transparent than the negative media (Supplementary Figure S6).

### 3.3. Overall Analysis of the Genome of DS-84

**Figure 3:**
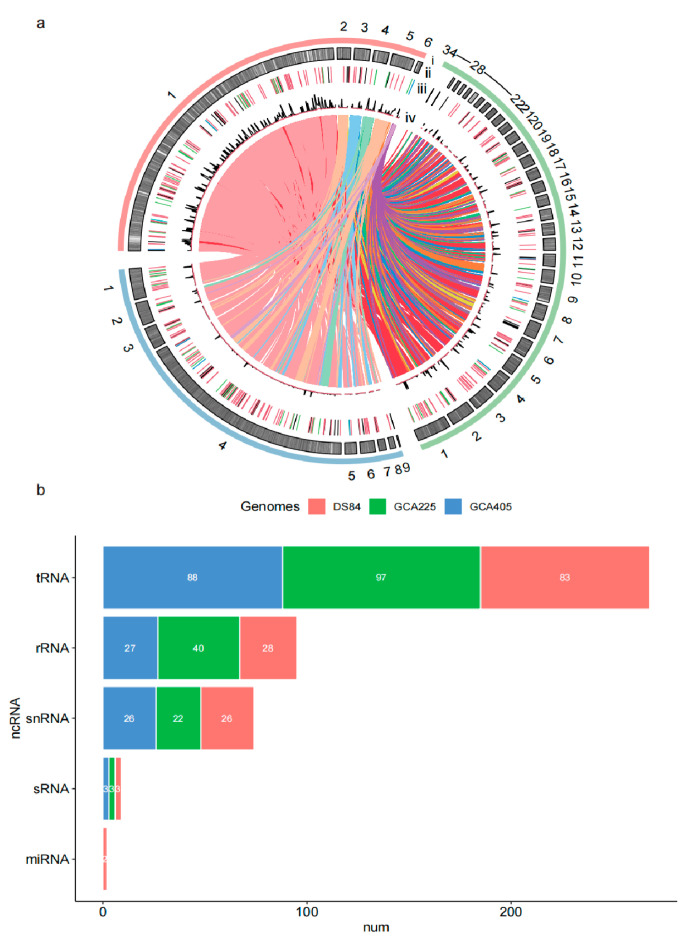
Chord diagram and ncRNA statistics among the three genomes. The three genomic compositions and the relationship between them are depicted by collinearity (**a**). track i: red is DS-84 genome, green is GCA225 and blue is GCA405; track ii: distribution of CDSs in three genomes; track iii: distribution of ncRNA in three genomes (light blue is miRNA, blue is sRNA, dark is snRNA, green is rRNA and red is tRNA); track iv: distribution of tandem repeats and interspersed repeats in three genomes (red is tandem repeats and dark is interspersed repeats). The non-coding RNAs are predicted in three genomes (**b**).

The genome of the *P. chrysanthemicola* DS-84 strain was sequenced using PacBio sequencing platforms. A total of 423,916 clean reads were obtained after correction. The clean reads were de novo assembled into 103 contigs with an N50 of 1.17 Mbp and an N90 of 0.27 Mbp (Table 3). All contings were further assembled into six scaffolds with a sequence length of 44,103,462 bp and N50 of 36.1 Mbp using reference genomes of *Paraphoma chrysanthemicola* Parch1 and *Paraphoma* sp. B47-9. The number of coding sequences (CDSs) from DS-84 was 37,077. DS-84′s genome length, N50 and CDS were larger than two others (Table 3). The chord diagram showed the vast amount of shared CDS sequences between DS-84 and the other two genomes (Figure 3a).

A total of 147 non-coding RNAs (ncRNAs) were predicted in the DS-84 genome, including 83 tRNAs, 28 rRNAs, 3 sRNAs, 26 snRNAs and 2 miRNAs. All ncRNAs were different in three *Paraphoma spp.* genomes (Figure 3b). The DS-84 gene had the fewest predicted tRNA sequences of the three genomes. The amount of predicted rRNAs in the DS-84 genome was only 70% of that in the GCA225 genome and similar to that in GCA405. The numbers of predicted snRNAs and sRNAs in the three genomes did not differ significantly. miRNAs were predicted only in the DS-84 genome.

Interspersed repeated sequences were counted for 9.57% of the DS-84 genome (Table 4). The majority of identifiable interspersed repeats were DNA transposons (2.72%), followed by long terminal repeat (LTR) elements (2.11%), and finally long interspersed repeated sequences (LINEs) (2.11%). The number and density of interspersed repetitive sequences in the DS-84 genome were the highest of the three genomes (Figure 3a). However, no short interspersed repeated sequences (SINEs) were predicted in all three genomes. The three genomes shared a similar density of tandem repeats with each other.

### 3.4. Functional Annotation of Genes in Three Genomes

**Figure 4:**
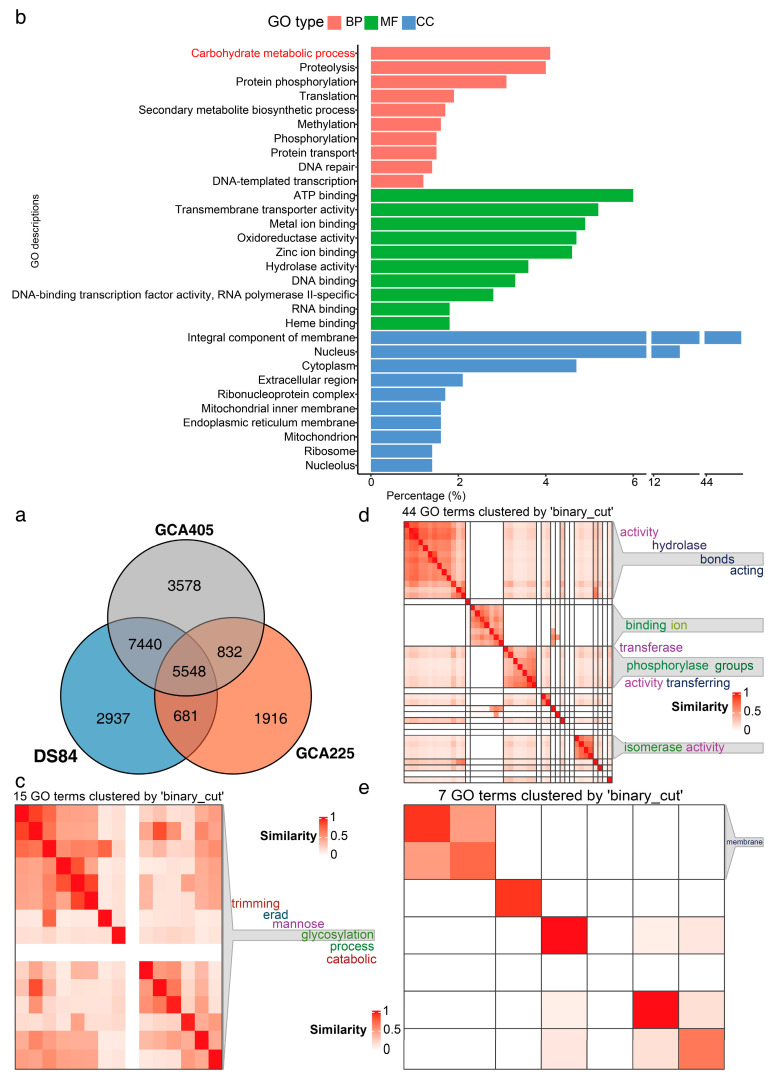
Common proteins of three genomes with GO annotation. Venn diagram analysis of common proteins in three genomes (**a**). Top ten three-class GO terms with the highest percentage of common GO annotations for the three genomes (**b**). GO terms of proteins clustered with semantic similarities involved in carbohydrate metabolism process (**c**): 15 GO terms of biological processes, (**d**): 44 GO terms of molecular functions, (**e**): 7 GO terms of cellular components.

Proteins identified using blast were annotated with GO in the uniprot database (Figure 4). The uniprot database annotation identified 5548 proteins common to all three genomes (Figure 4a). The GO terms of these 5548 proteins were counted and the top ten terms in percentage ranking are shown in Figure 4b. One of the top-ranked biological processes was the carbohydrate metabolism process. Further clustering similarity matrices of functional terms for the GO terms of proteins involved in the carbohydrate metabolism process showed that these proteins were mainly involved in glycosylation in mannose trimming of the endoplasmic reticulum (Figure 4c–e). We also performed KEGG enrichment analyses (Supplementary Figure S7). The three genomes were identical, with 365 pathways (Supplementary Figure S7a,b), 199 modules (Supplementary Figure S7c,d) and 702 reactions (Supplementary Figure S7e,f). The top ten pathways, modules and reactions included mainly carbon metabolism, N metabolism and RNA synthesis and metabolism.

### 3.5. Protein Advanced Annotation

**Figure 5:**
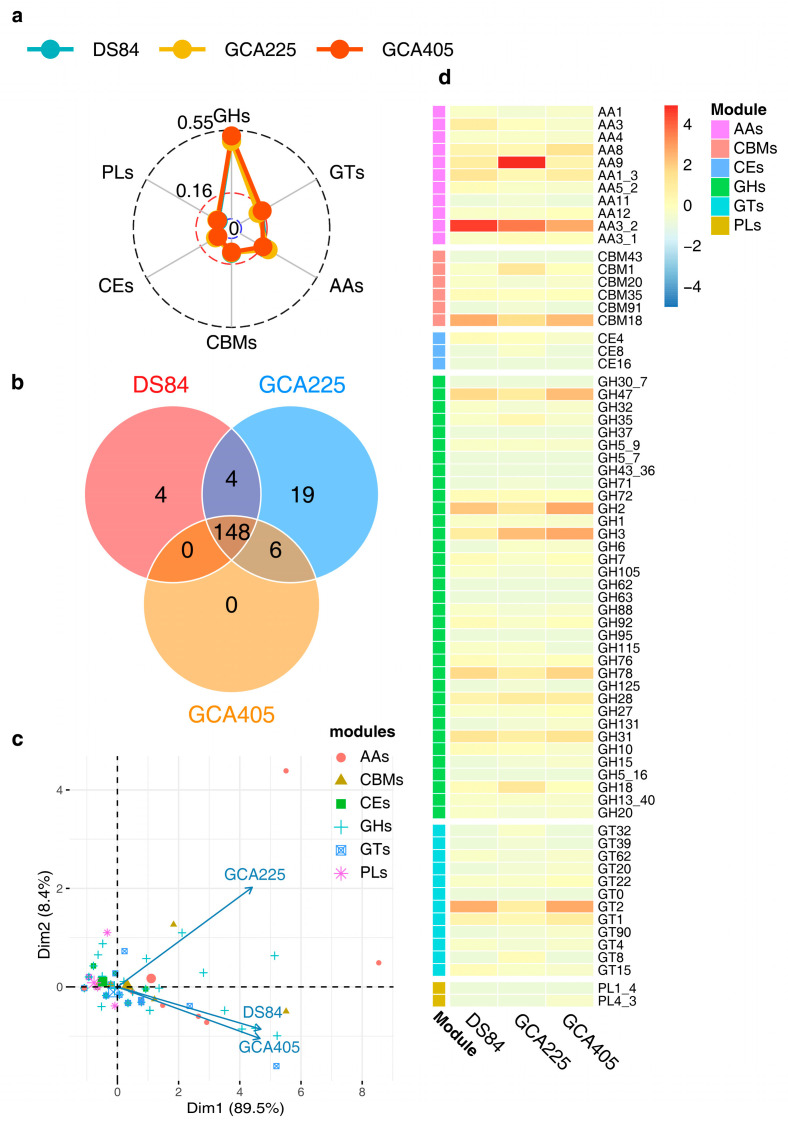
CAZy analysis of the three genomes. The radar chart depicts the proportion of different modules in the three genomes (**a**). Venn diagram showing modules shared by the three genomes (**b**). PCA of the correlation between different kinds of modules shared by the three genomes (**c**). Heatmap showing the common modules (number > 1) distributed among the three genomes (**d**).

The CAZy analysis of the three genomes revealed that the prediction ratios of different CAZy modules were similar in the three genomes, with the highest number of GH modules being DS-84 48.9%, GCA225 48.0% and GCA405 51.4% (Figure 5a). The number of shared modules in the three genomes was 148 (Figure 5b). Principal component analysis (PCA) of the 148 modules showed that, overall, the predicted number of different modules in the three genomes was positively correlated. This also indicated that the number of different types of modules was similarly distributed across the three genomes, with DS-84 and GCA495 having more similar numbers than GCA225. However, there were differences in the contents of specific types of modules in the three genomes, mainly auxiliary activities (AAs), carbohydrate-binding modules (CBMs), glycoside hydrolases (GHs) and glycosyltransferases (GTs). The above four groups of modules were more abundant in DS-84 and GCA495 than in GCA225 (Figure 5c). Combined with the heat map, AA3_2, CBMs18, GH2 and GT2 were predicted to be most abundant in DS-84 and GCA405, and AA9 was most abundant in GCA225 (Figure 5d) among the families themselves.

**Figure 6:**
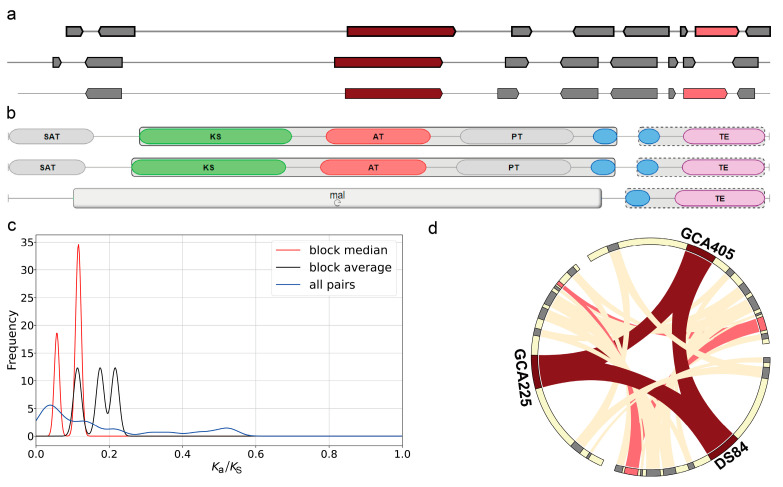
Biosynthetic gene clusters of melanin. Antismash was used to analyse the order of genes and the conserved domain of the core gene in melanin gene clusters (**a**,**b**). Ka/ks value depicting the genetic difference in melanin gene cluster (**c**). Chord diagram showing the collinearity of genes in melanin gene clusters (**d**), red: core biosynthetic gene, pink: additional biosynthetic gene, blue: transport-related gene, green: regulatory and grey: other genes.

Secondary metabolite-associated gene cluster analysis of the three genomes using the antiSMASH tool revealed three gene clusters that occurred simultaneously in the three genomes and had more than 60% similarity (Figure 6). The first one was the melanin biosynthetic gene cluster. The core biosynthetic genes in both DS-84 and GCA225 genomes contained the conserved domain of the N-terminal starter unit (SAT) (Figure 6b). The values of ka/ks were less than 0.25 and revealed the purifying selection of these clusters in three gene families (Figure 6c). Collinearity analysis revealed that additional biosyntheic gene (alcohol dehydrogenase) homologs in the DS-84 and GCA405 genomes were also present in the GCA225 genome (Figure 6d). The second was the dimethylcoprogen biosynthetic gene cluster (Supplementary Figure S8a). There was one core biosynthetic gene in this gene cluster. DS-84 had three additional biosynthetic genes, while GCA225 and GCA405 had four. DS-84 and GCA225 contained one transport-related gene and GCA405 contained two. The GCA225 genome in the core biosynthetic gene contained one more AMP-binding and peptidyl carrprotein (PCP) conserved domain compared with the DS-84 and GCA405 genomes (Supplementary Figure S8b). The ka/ks values between these genes were less than 0.2, synonymous changes predominated, and the sequence differences did not result in altered protein function (Supplementary Figure S8c). Collinearity analysis identified at least three additional biosynthetic genes and one transport-related gene that were homologous in the three genomes (Supplementary Figure S8d). The third was the phyllostictine A biosynthetic gene cluster (Supplementary Figure S6e–h). The gene clusters in the genus *Paraphoma* each had a core biosynthetic gene, a transport-related gene and a regulatory gene (Supplementary Figure S8e). There were four additional biosynthetic genes in the DS-84 and GCA225 genomes and five in the GCA405 genome. The core biosynthetic gene structure did not differ among the three genomes (Supplementary Figure S8f). The values of ka/ks among the three genomes were also less than 0.2, indicating a purifying selection (Supplementary Figure S8g). The collinearity analysis revealed that the core biosynthetic gene, additional biosynthetic genes, transport-related genes and regulatory gene were homologous among the three genomes, but the nucleotide distribution pattern of the core biosynthetic gene sequences changed in the GCA225 genome (Supplementary Figure S8h). Compared with the GCA405 genome, both the GCA225 and DS-84 genomes showed additional biosynthetic gene fusions.

## 4. Discussion

### 4.1. The Results Proved That DS-84 Is P. chrysanthemicola

*P. chrysanthemicola* is widely found in soil and can cause plant diseases or symbiosis with plants [16,19]. *P. chrysanthemicola* can cause leaf spot disease and root rot, as well as promote plant chlorophyll synthesis and increase plant resistance to salt and heavy metal stresses [14,17,19,21]. *P chrysanthemicola* was originally classified in the genus *Phoma,* but reassigned based on morphological and molecular characteristics [50,51]. Members of *Phoma* sect. *Paraphoma* were transferred to a range of genera, including *Paraphoma* [51]. *Ph. chrysanthemicola* was also renamed to *P. chrysanthemicola* [23]. *P. chrysanthemicola* can be characterised by its own morphology, FTIR absorption spectra and molecular phylogeny. Morphologically, the colonies of *P. chrysanthemicola* cultured in PDA medium were round or oval with irregular edges [17,21]. The colonies of *P. chrysanthemicola* over 2 weeks were dark grey or black and secreted brown–red pigment [21]. The microscopic morphology of the mycelium was observed with a diaphragm and the chlamydospore [52]. The chlamydospore may be unicellular or distributed in chains, globose or subglobose, with thick cell walls [14]. The phenotypes and micromorphologies observed in this study were consistent with those in previous studies on *P. chrysanthemicola*. At the same time, the results of electron microscopy further showed that the cell wall of *P. chrysanthemicola* was thick and there were abundant pores in it. It is possible that these pores were caused by longitudinal cross-linking of glucan and glycoprotein [53]. In addition to morphology, FTIR was used as an auxiliary tool for fungal identification. Previous studies have shown that there is little difference in the FTIR absorption peak characteristics of fungal strains before 2000 cm^−1^, but the absorption peaks between 1780–800 cm^−1^ are more different between different strains and can be used for strain identification [54]. The FTIR absorption spectra of the hyphal composition of members of the genus *Phoma* were divided into five strong peak regions, among which 1800–1485, 1485–1185 and 1185–900 cm^−1^ were the three sensitive spectral regions for analysis [55]. In this study, the FTIR absorption spectra of DS-84 in these three zones were similar to those of the genus *Phoma*, indicating that DS-84 was a close relative of the genus *Phoma.* Molecular phylogenetic analysis in the identification of *Paraphoma* sp. was quite important to avoid misidentifications via the above two approaches. The ITS region showed the highest probability of correct identification (PCI) for a large number of fungal pathogens with defined barcode gaps [56], while SSU and LSU were also very useful for species-level identification of fungal lineages [57]. The ITS, SSU and LSU sequences have been subjected to a phylogenetic analysis for the genus *Paraphoma* [23,58]. DS-84 was clustered with other known strains of the genus *Paraphoma* in the phylogenetic analyses and was highly polyphyletic within *P. chrysanthemicola* in a neighbour-joining tree. In conclusion, the results of identification and characterisation showed that *P. chrysanthemicola* was the answer to DS-84’s classification.

### 4.2. Monosaccharide Preference of P. chrysanthemicola

Research has shown that one of the monosaccharides preferred by fungi is mannose [59,60,61]. Mannose can be involved in catabolism or glycan biosynthesis [62]. Mannose is utilised by fungi as an energy source, and is also involved in the construction of polysaccharides and protein glycosylation [63,64]. A lack of mannose affects cell division, spore production and, thus, the normal life cycle, but excessive accumulation of mannose inhibits the synthesis of secondary metabolites [65,66,67,68]. The glycan biosynthesis of mannose in fungi requires two types of biological processes. One is the sequential actions of phosphomannose isomerase, phosphomannomutase and GDP-mannose pyrophosphorylase, which produces the mannose donor, GDP-mannose, in the cytosol [69,70]. GDP-mannose satisfies the requirement of mannose for oligosaccharides, glycoprotein and glycolipid synthesis to participate in the composition of fungal cell structure [69]. Another GH hydrolyzes the mannose residues in the host or its own oligosaccharides to release more mannose monomers [71,72]. This study found that mannose was the preferred monosaccharide to promote the weight of *P. chrysanthemicola*. The activation of cellulase has also been demonstrated in this study. Genome GO analysis revealed that the major genes related to the utilisation of mannose in *P. chrysanthemicola* were involved in mannose trimming. CAZy database analysis demonstrated that the enzyme classes of the carbohydrate-active enzymes were GHs at most in the *P. chrysanthemicola* genome. These results indicated that the mannose-related genes were essential for the maintenance of the morphology and normal physiological activities of *P. chrysanthemicola*.

### 4.3. The Important Secondary Metabolite Gene Cluster of P. chrysanthemicola

Fungi produce a large number of secondary metabolites to resist biotic and abiotic stresses [73]. Endophytic fungi improve the fitness of their host plants by directly producing antimicrobial and cytotoxic agents [74]. Most of the genes controlling fungal secondary metabolites are clustered together [75]. In this study, three important secondary metabolite gene clusters in the *P. chrysanthemicola* genome were predicted by antismash: melanin, dimethylcoprogen and phyllostictine A biosynthetic gene clusters. Melanin is a type of polyketide pigment, which can be synthesised via malonyl CoA, acetyl CoA, tyrosine or L-dopa [76]. Melanin promotes the resistance of fungi to radiation and, at the same time, allows fungi to adapt to the oxidative environment caused by the host’s immune response [76]. Dimethylcoprogen is a non-ribosomal peptide siderophore that promotes the utilisation of iron by fungi to enhance fungal growth and control the growth of other microorganisms in the environment [77]. Phyllostictine A has the ability to inhibit bacterial growth [78]. It has been shown that melanin could be extracted from the hyphae of *P. chrysanthemicola* [79]; the other two secondary metabolites have not been clearly reported in the genus *Paraphoma*. The colour change of *P. chrysanthemicola* in liquid medium indicated the synthesis of pigments, and genomic analysis showed that the colour change might be primarily affected by melanin. Conserved sequence analysis showed that melanin in *P. chrysanthemicola* was synthesised by acetyl-CoA. However, the presence of dimethylcoprogen and phyllostictine A synthesis gene clusters in the genome of *P. chrysanthemicola* was first reported. These metabolites help *P. chrysanthemicola* to become an endophytic fungus.

## 5. Conclusions

The endophytic fungi isolated from *Codonopsis* was *P. chrysanthemicola*. The results showed that *P. chrysanthemicola* had a mannose preference in monosaccharide utilisation, and melanin, dimethylcoprogen and phyllostictine A were important secondary metabolites.

## Figures and Tables

**Table 1:** Fatty acid composition of DS-84.

SN	Lipid Acid		RTS ^a^ (min)	RTSA ^b^ (min)	PAS ^c^ (pA·s)	RAP ^d^ (%)	CF ^e^	RFA ^f^ (%)
1	Palmitic Acid	C16:0	34.23	34.27	704.90	14.93	0.95	14.16
2	Palmitoleic Acid	C16:1	35.80	35.83	678.00	14.36	0.95	13.61
3	Stearic Acid	C18:0	39.39	39.44	153.30	3.25	0.95	3.10
4	Oleic Acid	C18:1n9c	41.09	41.22	1559.30	33.02	0.95	31.46
5	Linoleic Acid	C18:2ω6	43.96	44.07	1453.60	30.78	0.95	29.31
6	Eicosanoic Acid	C20:0	45.87	45.84	8.70	0.18	0.96	0.17
7	cis-11-Eicosenoic Acid	C20:1n9	47.67	47.69	32.90	0.7	0.96	0.67
8	Docosanoic Acid	C22:0	54.31	54.20	11.50	0.24	0.96	0.23
9	cis-4,7,10,13,16,19-Docosahexaenoic Acid	C22:6n3	76.32	76.26	7.20	0.15	0.96	0.14

^a^: Retention time of the standards. ^b^: Retention time of samples. ^c^: Peak area of samples. ^d^: Relative area percentage. ^e^: Conversion factor. ^f^: Relative content of individual fatty acid.

**Table 2:** Enzyme production capacity of strain DS-84.

Types of Enzymes	Enzyme Production Capacity
Amylase	+
Cellulase	+
Lipase	−
Alkaline protease	+
Fibrinolytic enzymes	−
Catalase	−
Chitosanase	+

“+” indicates a positive result; “−” indicates a negative result.

**Table 3:** Assembly statistics of three genomes.

Items	DS84	GCA225	GCA405
Total Length	44,103,462	40,663,325	38,237,961
Total Sequence Num	6	34	9
Total N Counts	8200	0	47,934
Total Low Case Counts	0	2,311,763	1,754,046
Total GC content	0.5	0.51	0.52
Minimum Length	551,857	8623	134,204
Maximum Length	36,058,779	3,887,370	24,712,919
Mean Length	7,350,577	119,980.15	4,248,662.33
Median Length	2,141,636.5	1,259,297	2,559,472
N50	36,058,779	1,781,809	24,712,919
CDS	37,077	15,088	36,812
Genome accession	GCA_030544205.1	GCA_020744225.13	GCA_001748405.1

The data showed the quality of the genome assembly. Raw data were obtained from the NCBI database. Raw data of DS84 and GCA405 were further assembled using Multi-CSAR.

**Table 4:** Statistics of interspersed repeated sequences in three genomes.

Interspersed Repeats	DS-84	GCA225	GCA405
Total interspersed repeats	9.57% (4,221,058 bp)	5.21% (2,116,650 bp)	1.56% (597,717 bp)
DNA transposons	2.72% (1,198,859 bp)	1.29% (525,828 bp)	0.26% (101,026 bp)
LTR elements	2.11% (932,269 bp)	1.77% (720,094 bp)	0.21% (81,527 bp)
LINEs:	0.23% (101,041 bp)	0.03% (11,678 bp)	0.01% (2178 bp)

## Data Availability

The whole-genome shotgun sequence used in this research can be found in https://www.ncbi.nlm.nih.gov (accessed on 9 October 2023). Genome accession is JARVTL000000000.1.

## Supplementary Materials

The following are available online at https://www.mdpi.com/article/10.3390/jof9101022/s1, Table S1: Primer sequence; Table S2: Reagents required for the experiment; Figure S1: Polymerase chain reaction-amplified ITS, SSU and LSU regions; Figure S2: Results of multiple ITS sequence alignments; Figure S3: Results of multiple SSU sequence alignments; Figure S4: Results of multiple LSU sequence alignments; Figure S5: Different sources of carbon, nitrogen and phosphorus gave different colours to the DS-84 colonies; Figure S6: Characterisation of the enzyme produced by the strain DS-84; Figure S7: Three genomes with common KEGG annotation; Figure S8: Biosynthetic gene clusters of dimethylcoprogen and phyllostictine A.
