# Supplementary material for: Genomic Characteristics and Comparative Genomics Analysis of the Endophytic Fungus Paraphoma chrysanthemicola DS-84 Isolated from Codonopsis pilosula Root
Source: J Fungi (Basel). 2023 Oct 16;9(10):1022. doi: 10.3390/jof9101022 (PMC10607767; doi:10.3390/jof9101022)
Supplement: Supplementary file 1 [file jof-09-01022-s001.zip › jof-2595723-supplementary.pdf]

Table S1. Primer sequence

Primer	Fragment	Primer sequences
ITS1	ITS1-5.8S-ITS2	5′-CTTGGTCATTTAGAGGAAGTAA-3′
ITS4		5′-TCCTCCGCTTATTGATATGC-3′
NS1	The partial small subunit	5′-GTAGTCATATGCTTGTCTC-3′
NS4	nuclear rDNA (SSU)	5′-CTTCCGTCAATTCCTTTAAG-3′
LROR	The partial large subunit	5′-ACCCGCTGAACTTAAGC-3′
LR5	nuclear rDNA (LSU)	5′-TCCTGAGGGAAACTTCG-3′

Table S2. Reagents required for the experiment[a]

Experiment	Name	Producer	Code
Scanning Electron Microscopy	Fixative for TEM	Servicebio	G1102
	Ethanol	Sinaopharm Group Chemical Reagent Co. LTD	100092183
	Isoamyl acetate	Sinaopharm Group Chemical Reagent Co. LTD	10003128
	PBS	Servicebio	G0002
	OsO4	Ted Pella Inc	18456
Genome	Fungi Genomic DNA Extraction Kit	Beijing Solabao Technology Co	D2300
	SMRTbell Express Template Preparation Kit 2.0	Pacific Biosciences	100-259-100
	NEBNext Ltra DNA Library Prep Kit	NEB	E7370L

a: The reagents for the other experiments were sourced from Sangon Biotech

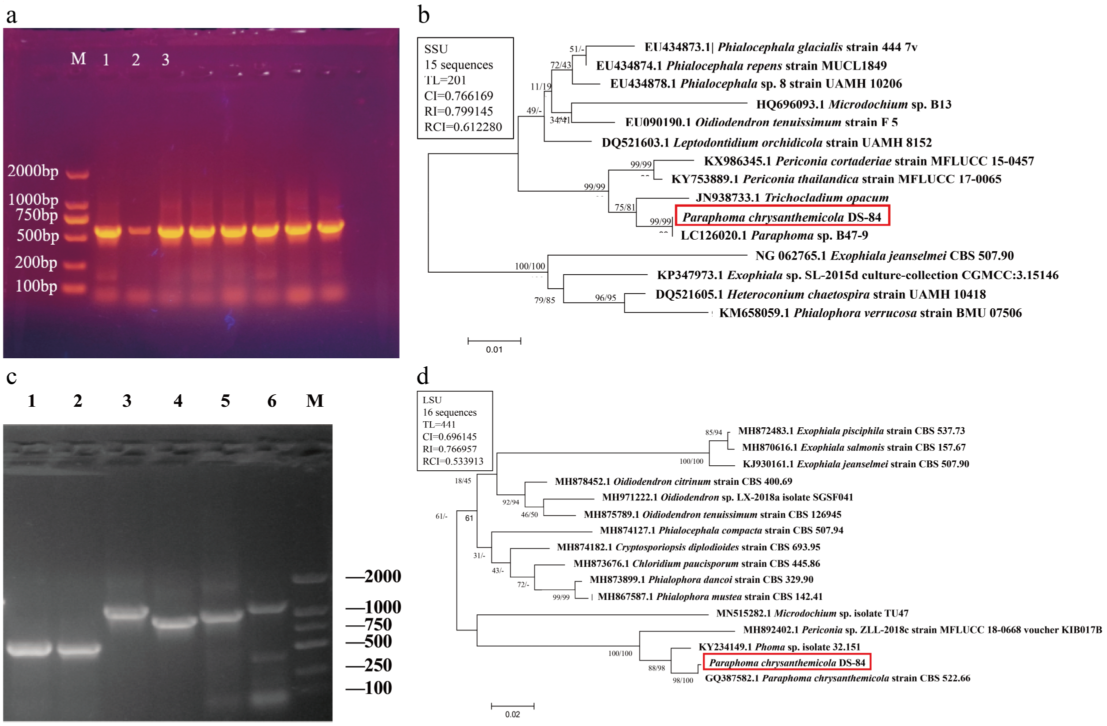

Supplementary Figure S1. Polymerase chain reaction amplified ITS, SSU and LSU region. a: Lane M was markers from 100 bp to 2000; lanes 1-3 were ITS results of strain DS-84 with three duplications. b: Lane M was markers from 100 bp to 2000; lanes 3, 5 were SSU results of strain DS-84; lanes 4, 6 were LSU results of strain DS-84. c: DS-84 phylogenetic tree was formed with 1000 bootstrap replicates based on SSU sequences. d: DS-84 phylogenetic tree was formed with 1000 bootstrap replicates based on LSU sequences. DS-84 has been highlighted with red boxes.

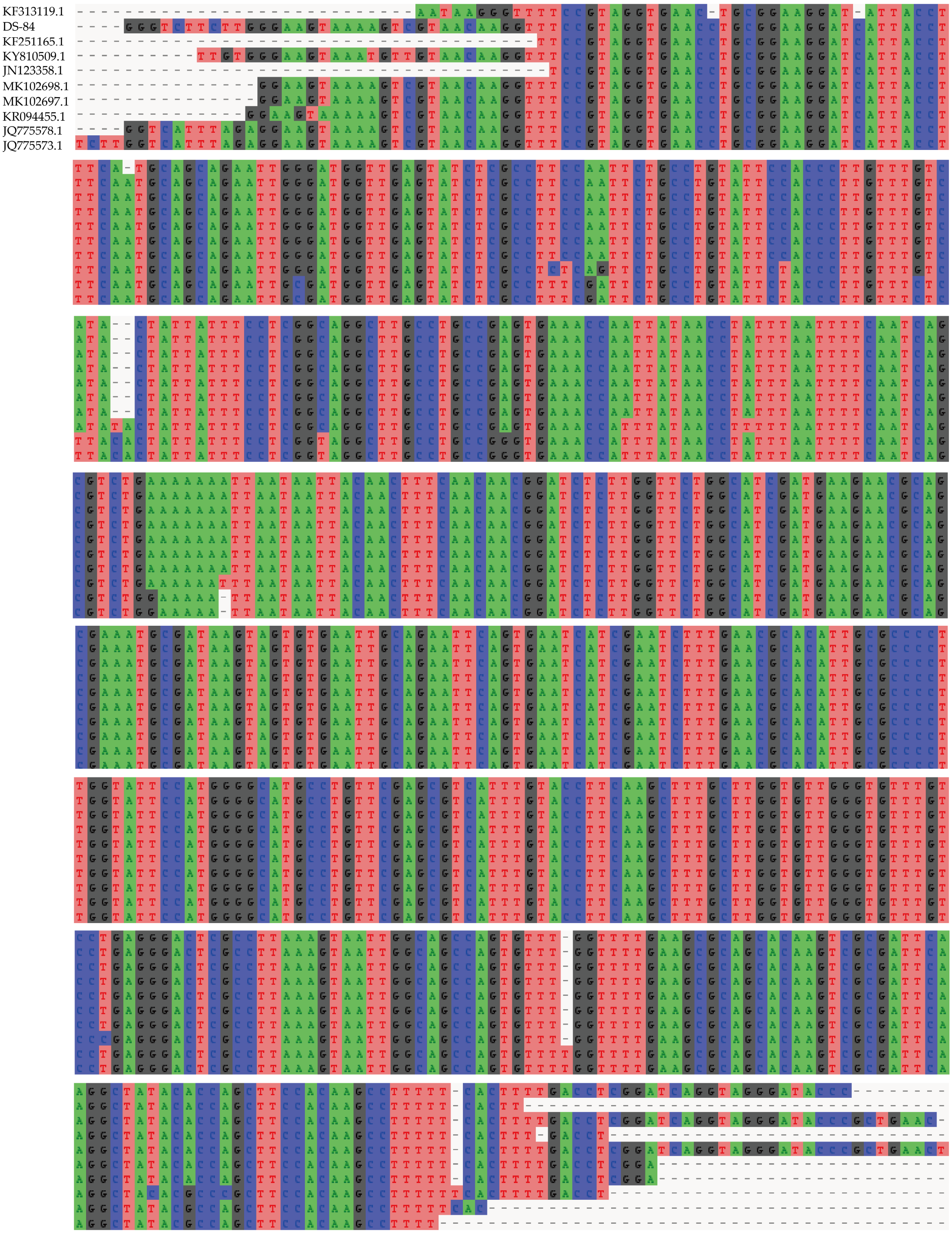

Supplementary Figure S2. The result of multiple ITS sequence alignments.

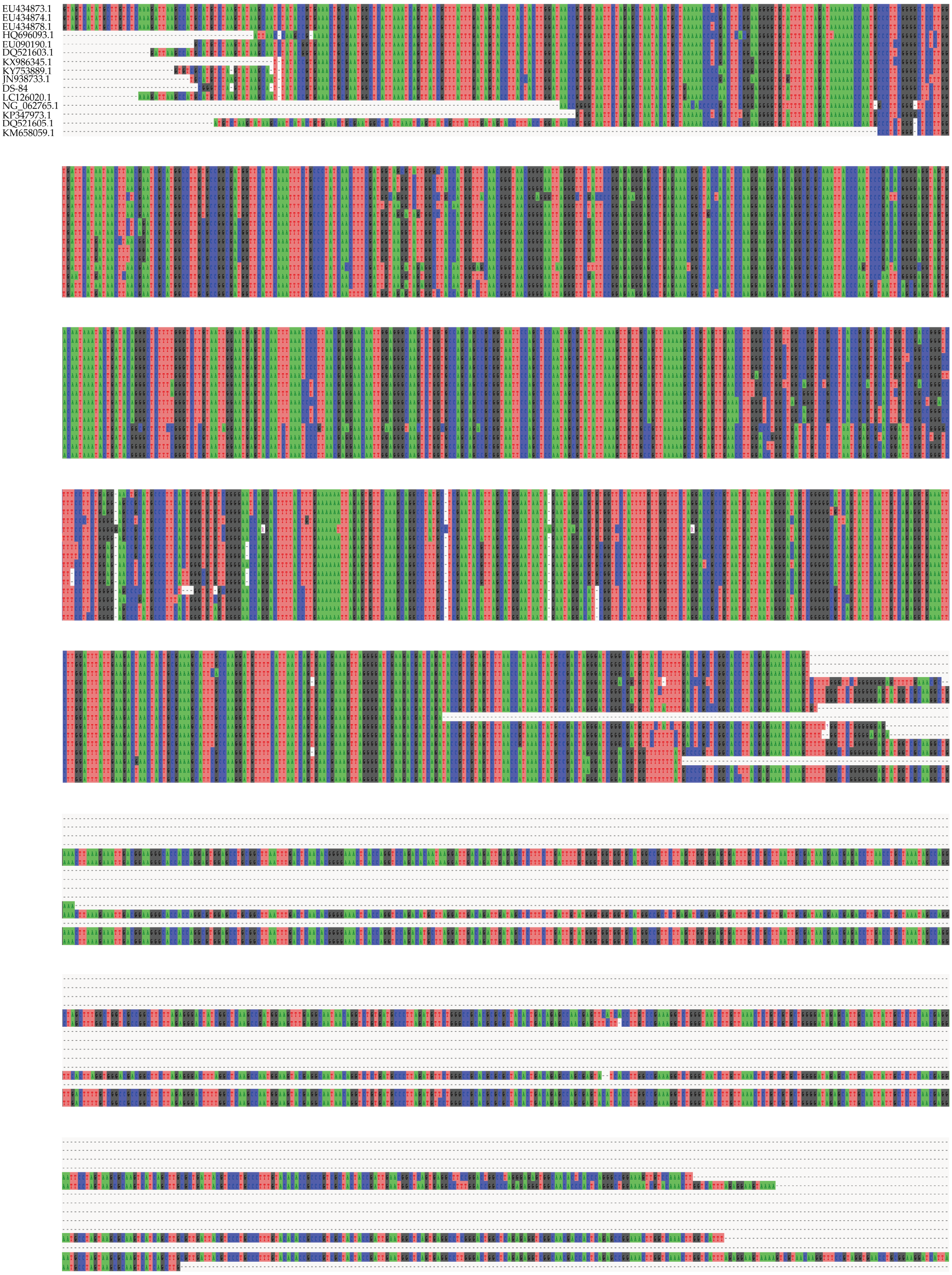

Supplementary Figure S3. The result of multiple SSU sequence alignments.

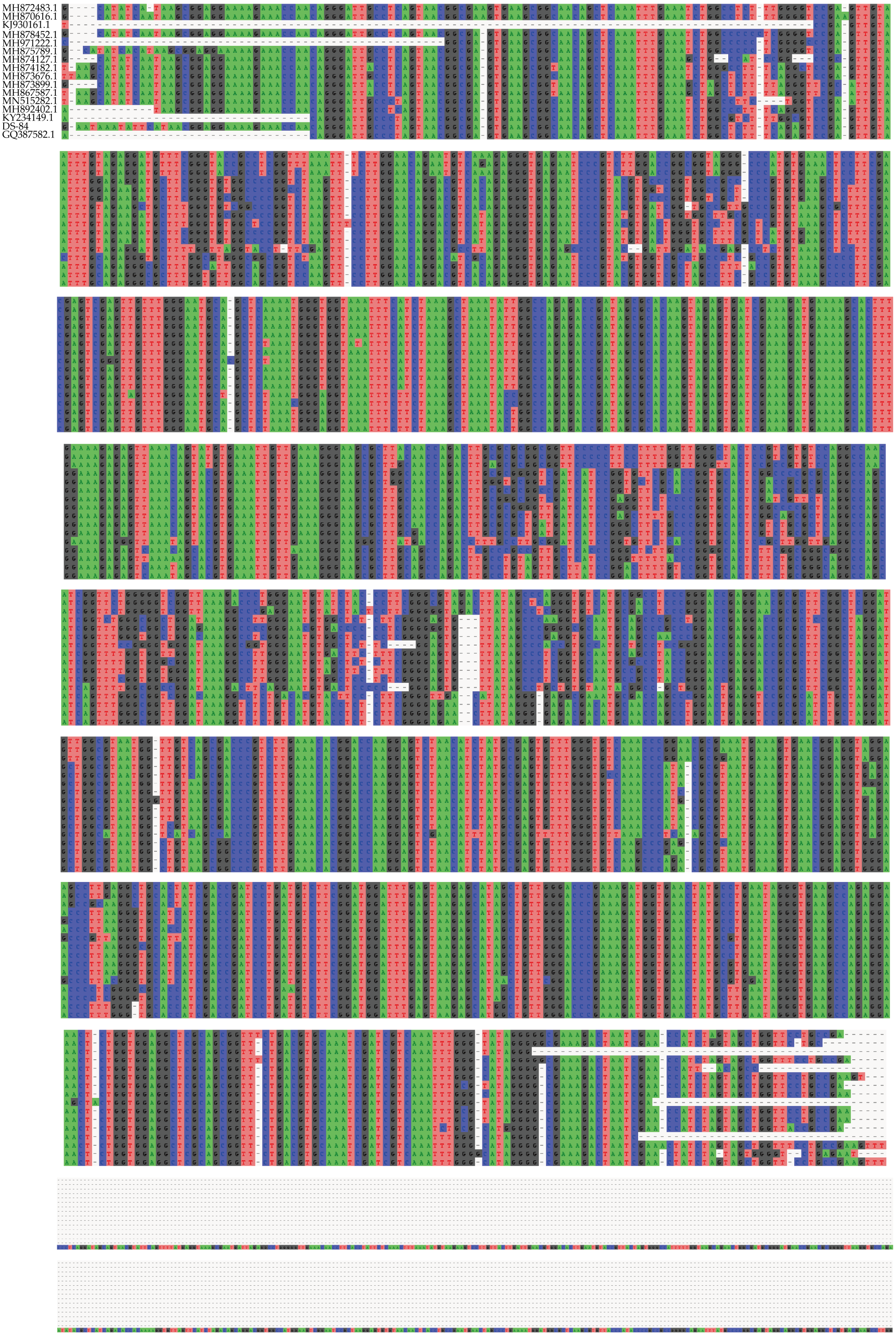

Supplementary Figure S4. The result of multiple LSU sequence alignments.

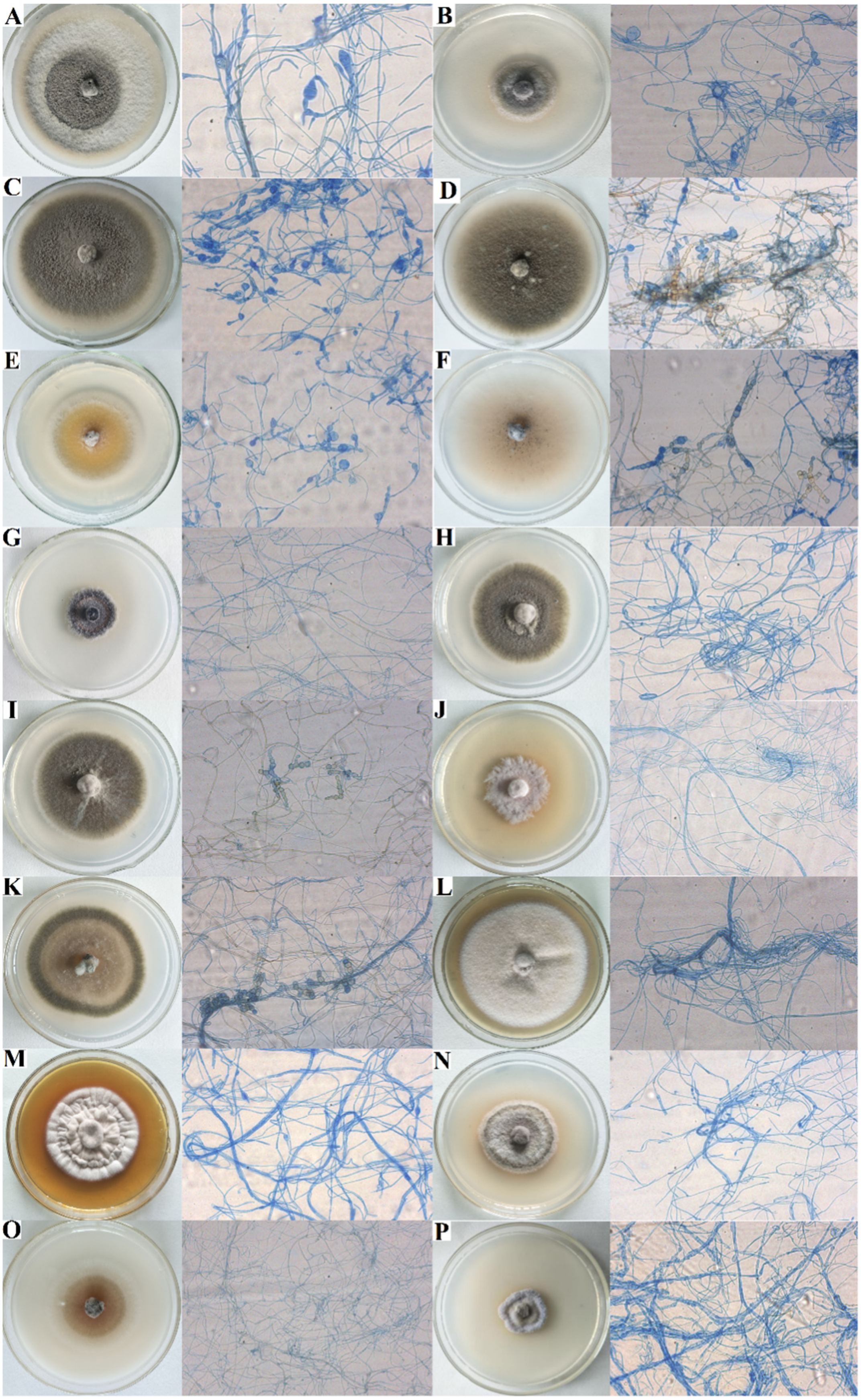

Supplementary Figure S5. Effects of different carbon, nitrogen and phosphorus sources on colony colour and mycelial morphology of strain DS-84. A: Glucose, B: Mannitol, C:

Fructose, D: Sucrose, E: Maltose, F: Lactose, G: Starch, H: $NaNO_3$, I: KNO_3, J: $(NH_4)_2SO_4$, K: $Ca(NO_3)_2$, L: Peptone, M: Yeast powder (YEP), N: KH_2PO_4, O: $Ca_3(PO_4)_2$, P: Lecithin.

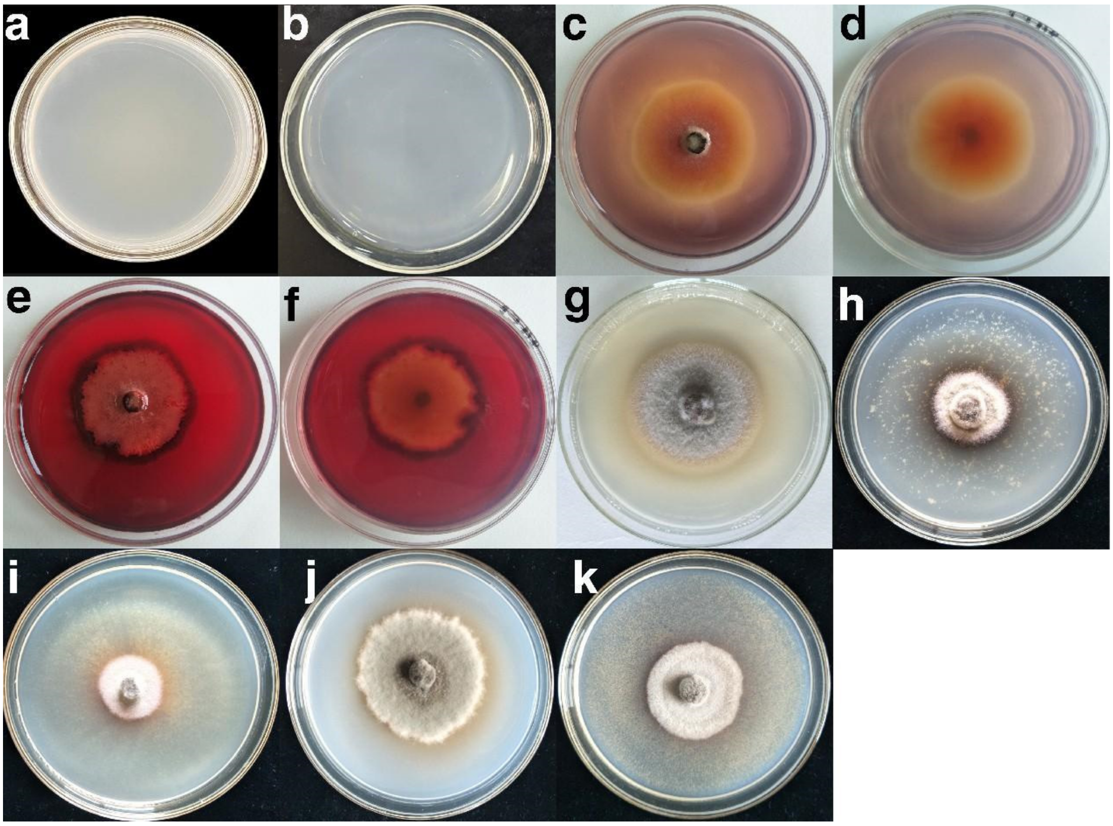

Supplementary Figure S6. Characterization of the enzyme produced by theStrain DS-84. Control (a: PDA, b: GYP), Amylase (c: front, d: back), Cellulase (e: front, f: back), Lipase (g), Alkaline protease (h), Fibrinolytic enzymes (i), Catalase (j), Chitosanase (k).

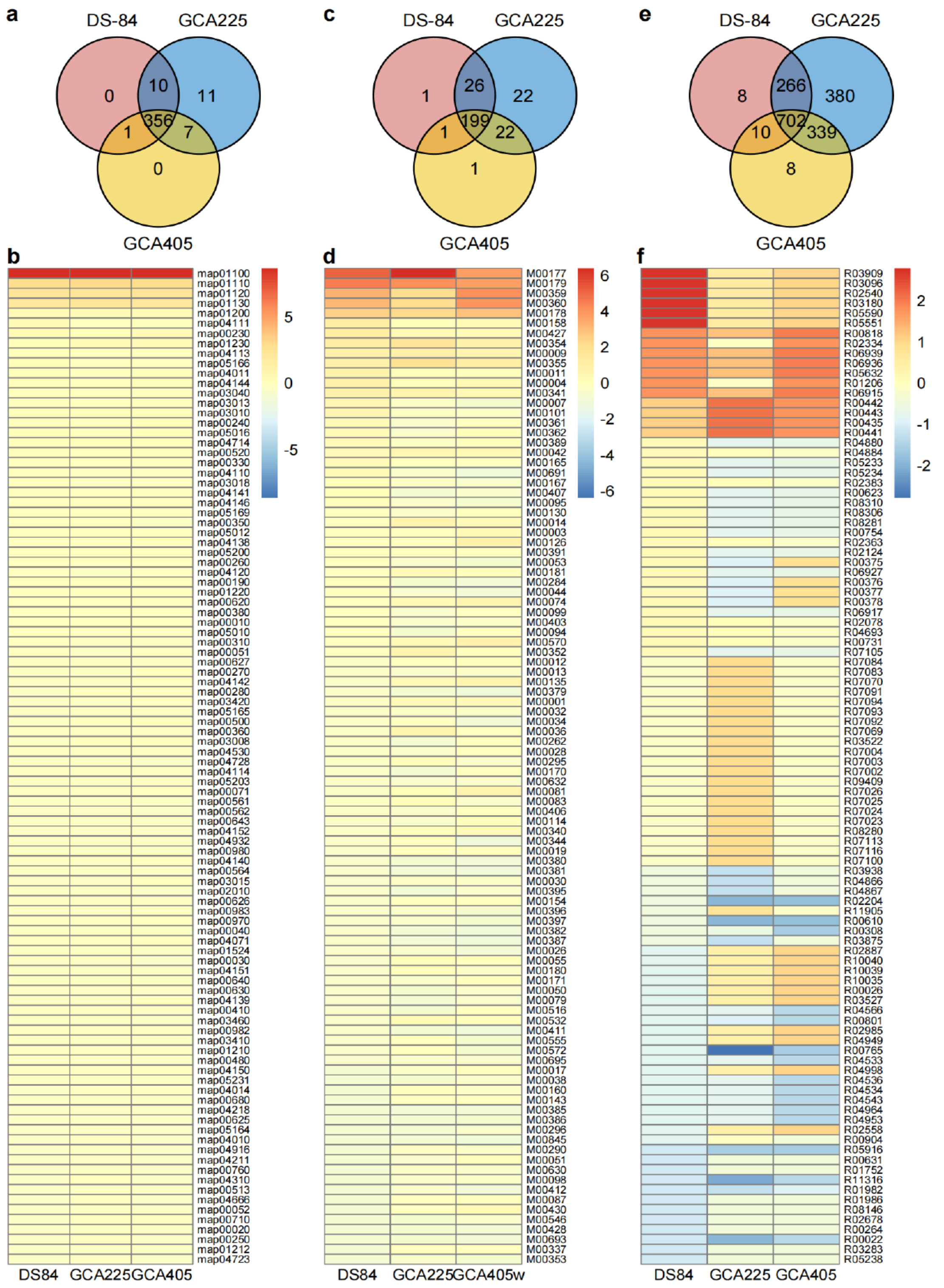

Supplementary Figure S7. Three genomes with common KEGG annotation. Venn showed the common ID of KEGG annotation, and heatmap depicted the proportion of different IDs (a-b: pathway ID, c-d: modules ID, e-f: reaction ID).

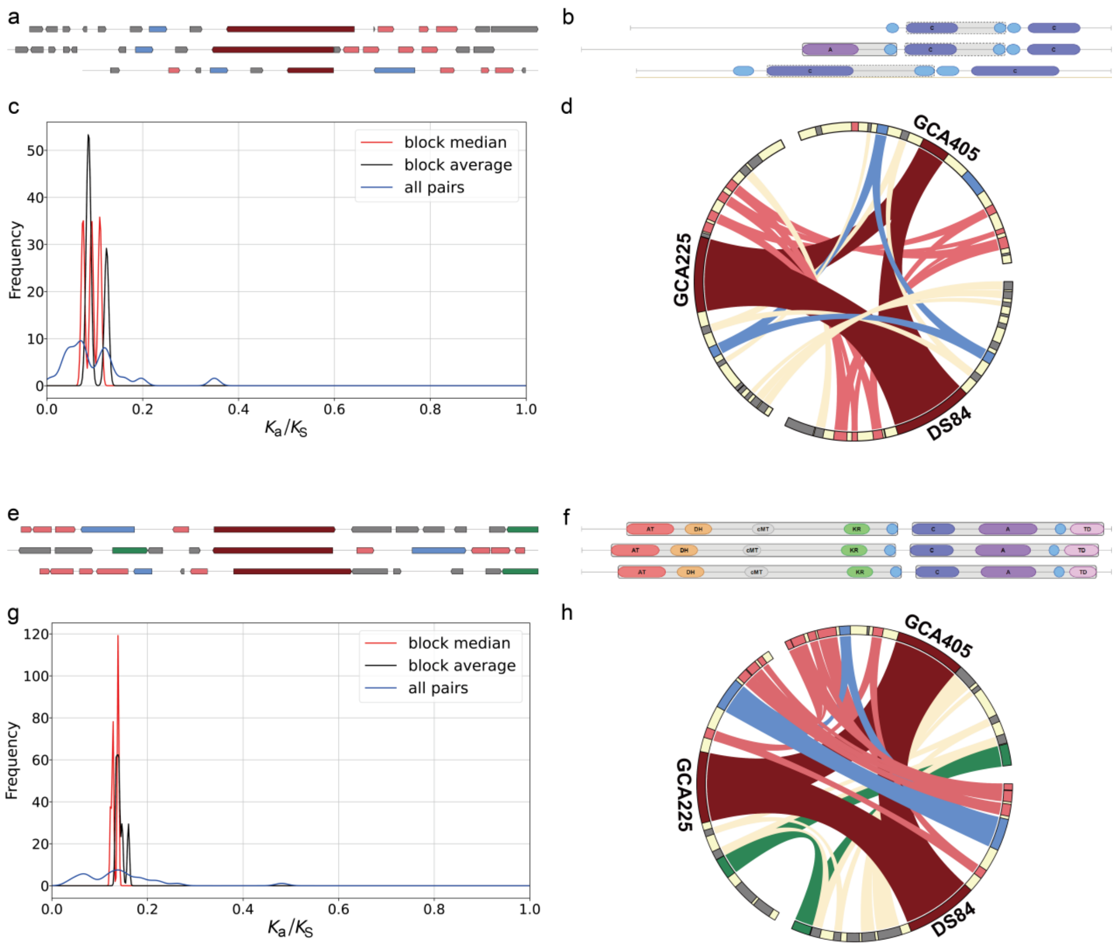

Supplementary Figure S8. Biosynthetic gene clusters of dimethylcoprogen and phyllostictine A. Antismash anaylzed the order of genes and conserved domain of core gene in other two gene clusters (a-b: dimethylcoprogen, e-f: phyllostictine). Ka/ks value depicted the genetic difference in two gene clusters (c: dimethylcoprogen, g: phyllostictine A). Chord diagram showed the colinearity of genes in two gene clusters (d: dimethylcoprogen, h: phyllostictine, red: core biosynthetic gene, pink: additional biosynthetic gene, blue: transport-related gene, green: regulatory, grey: other gene).
